# Redetermination of the distorted perovskite Nd_0.53_Sr_0.47_MnO_3_

**DOI:** 10.1107/S1600536808034168

**Published:** 2008-10-22

**Authors:** Ryoko Makita, Kiyoaki Tanaka, Masato Kubota, Youichi Murakami

**Affiliations:** a Graduate School of Materials Science & Engineering, Nagoya Institute of Technology, Gokiso-cho, Showa-ku, Japan; b Photon Factory, IMSS, High Energy Accelerator Research Organization (KEK), Tsukuba, 305-0801, Japan; c Materials Structure Physics Group, Department of Physics, Graduate School of Science, Tohoku University, Sendai 980-8578, Japan

## Abstract

Neodymium strontium manganese oxide with ideal composition Nd_0.5_Sr_0.5_MnO_3_ was reported to have two different structure models. In one model, the *x* coordinate of an O atom is at *x* > 1/2, while in the other model the *x*-coordinate of this atom is at *x* < 1/2. Difference-density maps around this O atom obtained from the current redetermination clearly show that the structure with the O atom at *x* < 1/2 result in a more satisfactory model than that with *x* > 1/2. The title compound with a refined composition of Nd_0.53 (5)_Sr_0.47 (5)_MnO_3_ is a distorted perovskite-type structure with site symmetries 2*mm* for the statistically occupied (Nd, Sr) site and for the above-mentioned O atom, .2/*m*. for the Mn atom and ..2 for a second O-atom site. In contrast to previous studies, the displacement factors for all atoms were refined anisotropically.

## Related literature

For details of the synthesis, see: Nakamura *et al.* (1999 ▶). For previous refinements of compounds with composition Nd_0.5_Sr_0.5_MnO_3_ from powder and single-crystal data, see: Woodward *et al.* (1998 ▶), Caignaert *et al.* (1998 ▶) and Kajimoto *et al.* (1999 ▶), Angappane *et al.* (2004 ▶), respectively. For general background, see: Becker & Coppens (1975 ▶); Dawson *et al.* (1967 ▶); Libermann *et al.* (1971 ▶); Mann (1968 ▶), Tanaka & Marumo (1983 ▶).

## Experimental

### 

#### Crystal data

Nd_0.53_Sr_0.47_MnO_3_
*M* *_r_* = 218.81
Orthorhombic, 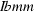

*a* = 5.4785 (3) Å
*b* = 5.4310 (3) Å
*c* = 7.6006 (5) Å
*V* = 226.14 (2) Å^3^
*Z* = 4
Mo *K*α radiation
μ = 28.37 mm^−1^
*T* = 241 (1) K
0.07 × 0.05 × 0.04 mm

#### Data collection

MAC Science M06XHF22 four-circle diffractometer
Absorption correction: numerical (*CCDABS*; Zhurov & Tanaka, 2003 ▶) *T*_min_ = 0.358, *T*_max_ = 0.521
1255 measured reflections
966 independent reflections
679 reflections with *F* > 3σ(*F*)
*R*_int_ = 0.022

#### Refinement

*R*[*F* ^2^ > 2σ(*F* ^2^)] = 0.028
*wR*(*F* ^2^) = 0.066
*S* = 1.19
927 reflections
65 parameters
14 restraints
Δρ_max_ = 2.17 e Å^−3^
Δρ_min_ = −3.38 e Å^−3^

### 

Data collection: *MXCSYS* (MAC Science, 1995 ▶) and *IUANGLE* (Tanaka *et al.*, 1994 ▶).; cell refinement: *RSLC-3 UNICS* system (Sakurai & Kobayashi, 1979 ▶); data reduction: *RDEDIT* (Tanaka, 2008 ▶); program(s) used to solve structure: *QNTAO* (Tanaka & Ōnuki, 2002 ▶; Tanaka *et al.*, 2008 ▶); program(s) used to refine structure: *QNTAO*; molecular graphics: *ATOMS for Windows* (Dowty, 2000 ▶); software used to prepare material for publication: *RDEDIT*.

## Supplementary Material

Crystal structure: contains datablocks global, I. DOI: 10.1107/S1600536808034168/wm2198sup1.cif

Structure factors: contains datablocks I. DOI: 10.1107/S1600536808034168/wm2198Isup2.hkl

Additional supplementary materials:  crystallographic information; 3D view; checkCIF report

## Figures and Tables

**Table 1:** Selected bond lengths (Å)

Mn—O1	1.9199 (6)
Mn—O2	1.9400 (4)
Nd^i^—O1^i^	2.501 (4)
Nd^ii^—O2	2.545 (2)
Nd^i^—O1	2.7332 (5)
Nd^ii^—O1	2.978 (4)

Symmetry codes: (i) 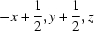; (ii) 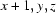.

**Fig. 1.**
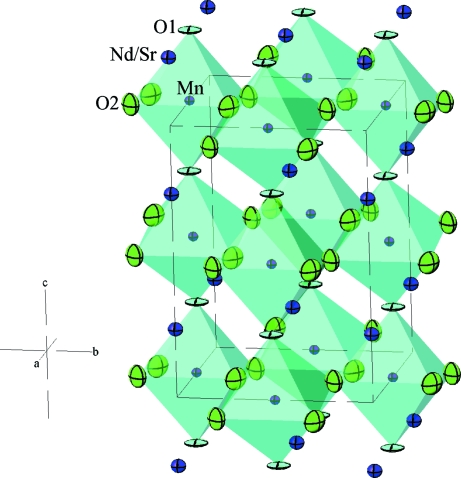
The structure of the distorted perovskite Nd0.53Sr0.47MnO3 with displacement ellipsoids drawn at the 90% probability level.

**Fig. 2.**
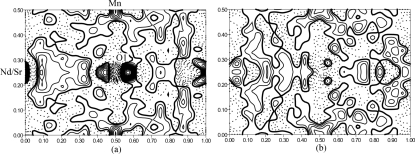
The difference density map projected onto (010) plane with a range 0 < x < 1 and 0< z < 1/2. (a): The x-coordinate of O1 is > 1/2. (b): The x-coordinate of O1 is < 1/2. Contours are given at intervals of 0.5 e Å-3. Zero contours are drawn as thick lines, positive contours are drawn as thin lines, and negative contours are drawn as broken line.

## supplementary crystallographic information

### Comment

Woodward *et al.* (1998) and Caignaert *et al.* (1998) determined the structure of Nd_0.5_Sr_0.5_MnO_3_ on the basis of powder X-ray diffraction data, whereas Kajimoto (1999) and Angappane *et al.* (2004) used single-crystal X-ray diffraction data for structure refinements. Except the model reported by Woodward *et al.* (1998), for all other structure models of Nd_0.5_Sr_0.5_MnO_3_ the *x*-coordinate of oxygen atom O1 was reported to be > 1/2. Since a new examination of the *x*-coordinate of O1 seemed desirable and anisotropic displacement factors were not reported in the previous studies, we decided to redetermine the structure of Nd_0.45_Sr_0.55_MnO_3_. The result of the structure analysis is presented in this communication.

The structure of the title compound derives from the perovskite-type (Fig. 1) and exhibits an orthorhombic distortion. The site symmetries are 2*mm* for the statistically occupied [(Nd,Sr)O_12_] polyhedron and for O1, .2/*m*. for the distorted [MnO_6_] octahedron and ..2 for O2.

### Experimental

A large single crystal was grown using a floating zone method (Nakamura *et al.*, 1999). The bulk sample was put on a piece of filter paper and was etched by diluted nitric acid under a microscope. Finally, the sample was shaped into a 0.040 mm × 0.053 mm × 0.065 mm block. Nd_0.45_Sr_0.55_MnO_3_ exhibits a first order phase transition at T_N_ = 225K. The present diffraction study was carried out at 241 (1) K close to the phase transition temperature.

### Refinement

The structure of Nd_1 -_*_x_*Sr*_x_*MnO_3_ changes with the hole-concentration *x*. In the region *x* > 0.55 the structure has tetragonal symmetry. However, at *x* = 0.60 a phase with monoclinic symmetry was reported at low temperature (Kajimoto *et al. *, 1999). Below *x* = 0.55 it changes to orthorhombic symmetry. Since crystals with monoclinic, orthorhombic and tetragonal symmetries were found in preliminary experiments for crystal with approximate compositions of Nd_0.45_Sr_0.55_MnO_3_ (which is expected from the composition of the starting materials), the hole-concentration *x* (i.e. site occupation factors) were also refined besides the atomic coordinates and the temperature factors. The previous studies (Woodward *et al.* (1998); Caignaert *et al.* (1998); Angappane *et al.* (2004)) have used the standard setting of space group No. 74 in *Imma*. We decided to refine the structure with the setting in *Ibmm*, because in the orthorhombic phase the crystal axis is taken along the same direction as that of the tetragonal phase which is also adopted by many other physicists to make clear the relationships between the two phases. Furthermore, Nd_0.45_Sr_0.55_MnO_3_ is well known as having dx^2^-y^2^- type orbital-ordering of Mn and the physical and chemical properties are discussed based on the *Ibmm* setting.

When the coordinates by Caignaert *et al.* (1998) were used as starting parameters for refinement, the *x*-coordinate of O1 converged to 0.518 (1) with a *R*-factor of 0.0381. Fig. 2 (*a*) shows the difference density map onto (010) after this refinement in the range 0< *z* < 1/2 and 0 < *x* < 1 with the vertical and horizontal lengths of 3.80 Å × 5.48 Å. The cores of Nd/Sr, Mn and O1 are at (0, 1/4), (1/2, 1/2) and (0.52, 1/4). Since there are two high peaks at *x* = 0.45 and 0.55 in Fig 2 (*a*), O1 was split into O1(1) at *x*=0.45 and O1(2) at *x*=0.55. The site occupation factors of O1(1) and O1(2) became 0.96 (6) and 0.04 (6) after the refinement. Hence O1 was concluded to be located only at *x*=0.45. After the subsequent refinement the *R*-factor converged at 0.0289 and the difference density map became likewise more satisfactory (Fig. 2(*b*)).

Although the temperature factor U^33^ of O1 is 0.001 (1) Å^2^ and almost insignificant, it becomes 0.0015 (2) Å^2^ after the refinement of anharmonic vibration parameters (Dawson *et al.*, 1967; Tanaka & Marumo, 1983) as well as the harmonic ones. Finally, refinement of the site occupation factors revealed a hole-concentration *x* of 0.47 (5) thus leading to a composition of Nd_0.53_Sr_0.47_MnO_3_.

### Crystal data

**Table:**

Nd_0.53_Sr_0.47_MnO_3_	*F*(000) = 394.64
*M**_r_* = 218.81	*D*_x_ = 6.479 Mg m^−^^3^
Orthorhombic, *I**b**m**m*	Mo *K*α radiation, λ = 0.71073 Å
Hall symbol: -I 2c 2c	Cell parameters from 30 reflections
*a* = 5.4785 (3) Å	θ = 35.6–37.8°
*b* = 5.4310 (3) Å	µ = 28.37 mm^−^^1^
*c* = 7.6006 (5) Å	*T* = 241 K
*V* = 226.14 (2) Å^3^	Block, black
*Z* = 4	0.07 × 0.05 × 0.04 mm

### Data collection

**Table:**

MAC Science M06XHF22 four-circle diffractometer	966 independent reflections
Radiation source: fine-focus rotating anode	679 reflections with *F* > 3σ(*F*)
graphite	*R*_int_ = 0.022
Detector resolution: 1.25x1.25° pixels mm^-1^	θ_max_ = 74.7°, θ_min_ = 5.3°
integrated intensities data fom ω/2θ scans	*h* = −12→14
Absorption correction: numerical (CCDABS; Zhurov & Tanaka, 2003)	*k* = −12→14
*T*_min_ = 0.358, *T*_max_ = 0.521	*l* = −18→18
1255 measured reflections	

### Refinement

**Table:**

Refinement on *F*	14 restraints
Least-squares matrix: full	Weighting scheme based on measured s.u.'s
*R*[*F*^2^ > 2σ(*F*^2^)] = 0.028	(Δ/σ)_max_ = 0.0002
*wR*(*F*^2^) = 0.066	Δρ_max_ = 2.17 e Å^−^^3^
*S* = 1.19	Δρ_min_ = −3.38 e Å^−^^3^
927 reflections	Extinction correction: B–C type 1 Gaussian anisotropic (Becker & Coppens, 1975)
65 parameters	Extinction coefficient: 0.029E04 (1)

### Special details

**Table:**

Experimental. Multiple diffraction was avoided by ψ-scan. Intensities was measured at equi-temperature region of combinaion of angles ω and χ of four-circle diffractometer
Refinement. B—C anisotropic type1 extinction parameters (× 10 ^4^s) are as follows 4087 (526) 6631 (1159) 3088 (391) -790 (416) -1835 (361) 3716 (625)Dawson *et al.* (1967) proposed the treatment of temperature factors including anharmonic thermal vibration (AHV) effect for high-symmetry crystals by means of series expansion of an one-particle-potential. Tanaka and Marumo (1983) generalized the treatment and anharmonic third and fourth order parameters were refined in the least-square program. AHV parameters were restricted by the site symmetry of Nd/Sr(2 mm), Mn(.2/m.), O1(2 mm) and O2(..2). The anharmonic potentials (V) are represented by the following equation:V_Nd,Sr,O1_=c_111_u_1_^3^+c_123_u_1_u_2_^2^+c_133_u_1_u_3_^2^+q_1111_u_1_^4^ +q_1122_u_1_^2^u_2_^2^+q_1133_u_1_^2^u_3_^2^+q_2222_u_2_^4^ +q_2233_u_2_^2^u_3_^2^+q_3333_u_3_^4^ ···(1)V_Mn_=q_1111_u_1_^4^+q_1122_u_1_^2^u_2_^2^+q_1133_u_1_^2^u_3_^2^ +q_2222_u_2_^4^+q_2233_u_2_^2^u_3_^2^+q_3333_u_3_^4^+q_1131_u_1_^3^u_3_ +q_2231_u_2_^2^u_1_u_3_+q_3331_u_3_^3^u_1_ ···(2)V_O2_=c_211_u_1_^2^u_2_+c_222_u_2_^3^+c_233_u_3_^2^u_2_+c_123_u_1_u_2_u_3_ +q_1111_u_1_^4^+q_1122_u_1_^2^u_2_^2^+q_1133_u_1_^2^u_3_^2^ +q_2222_u_2_^4^+q_2233_u_2_^2^u_3_^2^+q_3333_u_3_^4^+q_1131_u_1_^3^u_3_ +q_2231_u_2_^2^u_1_u_3_+q_3331_u_3_^3^u_1_ ···(3)where (u_1_,u_2_,u_3_) is a displacement vector from equilibrium position of each atom. The displacement vector of Nd, Sr, O1 was defined on the coordinate system with axes parallel to the crystal axes, a, b and c. That of Mn and O2 was defined by equation (4) and (5) in terms of the lattice vectors a, b and c in the present study.u_1_= -0.18253*a*, u_2_= 0.18413*b*, u_3_= -0.13157*c* ···(4)u_1_= -0.11080*a*-0.14633*b*, u_2_= 0.13157*c*, u_3_= -0.14506*a* + 0.11177*b* ···(5)Since there is strong correlation between harmonic temperature factors and AHV parameters, the AHV parameters and the harmonic temperature factors were refined alternately. The significant AHV parameters c_ijk_ (× 10^-19^JÅ^-3^) and q_iijk_ (× 10^-19^JÅ^-3^) are as follows:Nd and Sr; c_111_= -5.9 (49), c_122_= -3.8 (14),Mn; q_2231_= -1832 (1560),O1: q_2222_= -9.5 (39), q_2233_= 569.9 (2279),O2: c_211_= 3.7 (33), c_233_= 0.8 (7), c_123_= -5.5 (23), q_2233_= 9.1 (79),

### Fractional atomic coordinates and isotropic or equivalent isotropic displacement parameters (Å^2^)

**Table:**

	*x*	*y*	*z*	*U*_iso_*/*U*_eq_	Occ. (<1)
Nd	−0.00656 (9)	0	0.25	0.00637 (4)	0.53 (5)
Sr	−0.00656 (9)	0	0.25	0.00637 (4)	0.47 (5)
Mn	0.5	0	0	0.00305 (7)	
O1	0.4499 (8)	0	0.25	0.0112 (6)	
O2	0.75	0.25	0.0276 (4)	0.0139 (5)	

### Atomic displacement parameters (Å^2^)

**Table:**

	*U*^11^	*U*^22^	*U*^33^	*U*^12^	*U*^13^	*U*^23^
Nd	0.00653 (6)	0.00685 (7)	0.00574 (8)	0	0	0
Sr	0.00663 (6)	0.00685 (7)	0.00574 (8)	0	0	0
Mn	0.0035 (1)	0.0030 (1)	0.0027 (1)	0	0	0
O1	0.015 (1)	0.017 (1)	0.0015 (2)	0	0	0
O2	0.0148 (7)	0.0116 (7)	0.015 (1)	−0.0058 (6)	0	0

### Geometric parameters (Å, °)

**Table:**

Mn—O1	1.9199 (6)	Nd^ii^—O2	2.545 (2)
Mn—O2	1.9400 (4)	O1—O2	2.721 (3)
Nd^i^—Nd^ii^	3.8064 (5)	Nd^i^—Mn	3.3043 (4)
Nd^ii^—O1^ii^	2.501 (4)	O1^iii^—O2^ii^	2.738 (3)
Nd^i^—O2	2.545 (2)	O1—O1^ii^	3.489 (4)
Nd^ii^—O1	2.7332 (5)	O2—O2^iii^	3.8799 (5)
Nd^i^—O1	2.978 (4)		
			
Nd^ii^—Nd^i^—Mn	55.020 (8)	Nd^i^—O1—Mn	81.8 (1)
Mn—Nd^i^—O1	35.10 (8)	Nd^ii^—O1—Mn	89.07 (3)
Nd^i^—Nd^ii^—Mn	54.769 (6)	Mn—O1—O1^ii^	95.15 (9)
Nd^i^—Nd^ii^—O2	41.61 (5)	Nd^ii^—O1^ii^—O1	51.11 (8)
Mn—Nd^ii^—O1^ii^	89.4 (1)	Nd^i^—O2—Nd^ii^	96.8 (1)
O1—Nd^ii^—O1^ii^	83.5 (1)	Nd^i^—O2—O2^iii^	144.59 (7)
O1^ii^—Nd^ii^—O2	121.6 (1)	Nd^ii^—O2—O2^iii^	47.83 (1)
Nd^i^—Mn—Nd^ii^	70.212 (7)	Nd^ii^—Nd^i^—O2	41.61 (5)
Nd^ii^—Mn—O1	55.54 (2)	O1—Nd^i^—O2	58.4 (1)
Nd^i^—O1—Nd^ii^	83.48 (9)	Nd^i^—Nd^ii^—O1^ii^	134.5 (1)
Nd^i^—O1—O2	52.83 (8)	Mn—Nd^ii^—O1	35.39 (1)
Nd^ii^—O1—O2	55.64 (6)	Mn—Nd^ii^—O2^iii^	87.76 (5)
O1^ii^—O1—O2	89.48 (6)	O1—Nd^ii^—O2^iii^	114.44 (5)
O1—O1^ii^—O2^iii^	97.8 (1)	O2—Nd^ii^—O2^iii^	91.19 (7)
Nd^i^—O2—O1	68.77 (9)	Nd^i^—Mn—O2	50.22 (1)
Nd^ii^—O2—O1	62.42 (8)	Nd^i^—O1—O1^ii^	128.89 (6)
Nd^ii^—Nd^i^—O1	45.51 (8)	Nd^ii^—O1—O1^ii^	45.41 (5)
Mn—Nd^i^—O2	35.85 (5)	Mn—O1—O2	45.48 (6)
Nd^i^—Nd^ii^—O1	51.01 (1)	Nd^ii^—O1^ii^—O2^iii^	66.4 (1)
Nd^i^—Nd^ii^—O2^iii^	132.79 (5)	Nd^i^—O2—Mn	93.92 (6)
Mn—Nd^ii^—O2	35.71 (5)	Nd^ii^—O2—Mn	94.32 (6)
O1—Nd^ii^—O2	61.94 (5)	Mn—O2—O2^iii^	88.84 (2)
O1^ii^—Nd^ii^—O2^iii^	60.7 (1)	O1—O2—O2^iii^	89.42 (6)
Nd^i^—Mn—O1	63.12 (2)	O1^ii^—O2^iii^—O2	81.49 (5)
Nd^ii^—Mn—O2	49.98 (1)	Nd^ii^—O2^iii^—O1^ii^	52.84 (6)

Symmetry codes:  (i) *x*+1, *y*, *z*; (ii) −*x*+1/2, *y*+1/2, *z*; (iii) *x*−1/2, *y*+1/2, −*z*.
